# Prediction of high-risk areas for visceral leishmaniasis using socioeconomic indicators and remote sensing data

**DOI:** 10.1186/1476-072X-13-13

**Published:** 2014-05-20

**Authors:** Andréa S Almeida, Guilherme L Werneck

**Affiliations:** 1 Instituto de Medicina Social, Universidade do Estado do Rio de Janeiro, Rua São Francisco Xavier, 524, Pavilhão João Lyra Filho, 7º andar/blocos D e E, e 6º andar/bloco E, Maracanã, CEP 20550-013 Rio de Janeiro, Brazil; 2 Instituto de Estudos em Saúde Coletiva, Universidade Federal do Rio de Janeiro, Avenida Horácio Macedo, S/N - Próximo a Prefeitura Universitária da UFRJ, Ilha do Fundão - Cidade Universitária, CEP 21941-598 Rio de Janeiro, RJ, Brazil

**Keywords:** Leishmaniasis, Predictive models, Remote sensing

## Abstract

Spatial heterogeneity in the incidence of visceral leishmaniasis (VL) is an important aspect to be considered in planning control actions for the disease. The objective of this study was to predict areas at high risk for visceral leishmaniasis (VL) based on socioeconomic indicators and remote sensing data. We applied classification and regression trees to develop and validate prediction models. Performance of the models was assessed by means of sensitivity, specificity and area under the ROC curve. The model developed was able to discriminate 15 subsets of census tracts (CT) with different probabilities of containing CT with high risk of VL occurrence. The model presented, respectively, in the validation and learning samples, sensitivity of 79% and 52%, specificity of 75% and 66%, and area under the ROC curve of 83% and 66%. Considering the complex network of factors involved in the occurrence of VL in urban areas, the results of this study showed that the development of a predictive model for VL might be feasible and useful for guiding interventions against the disease, but it is still a challenge as demonstrated by the unsatisfactory predictive performance of the model developed.

## Introduction

Since the 1980s, visceral leishmaniasis (VL) had its epidemiological profile modified in Brazil; no longer being characterized as a predominantly rural disease, but established in the urban environment as well [1].

The introduction, propagation and dissemination of VL in urban settings is associated to multiple and complex conditions, such as environmental changes due to migration movements, disorderly occupation of city’s outskirts, high population density, and inadequate living conditions [2,3].

The spatial distribution of urban VL is markedly heterogeneous, which may lead to a substantial increase in transmission levels [3]. In this situation, focusing intervention on high risk areas might be an efficient strategy to reduce transmission rates [4].

The objective of the present study is to characterize and predict high risk areas for the occurrence of VL in Teresina, Piauí, based on socioeconomic indicators and environmental data, obtained through remote sensing.

## Methods

### Area of study

Teresina, capital of Piauí state, in the northeast region of Brazil, is located between the confluence of the rivers Parnaíba e Poti, at a 5°5′ south latitude and 42°48′ west longitude. Teresina has a population of 793,915 inhabitants and population density of 444.2 inhabitants/km [2]. It has a tropical sub-humid climate, with high registered temperatures throughout the year, varying from 22°C to 40°C. Rainy season is mainly from January until April.

### Study design

This is an ecologic study, in which the units of analysis are the 430 and 653 urban census tracts (CT) of Teresina for the years of 1991 and 2000, respectively.

### Data and variables

#### Response variable

**Figure 1:**
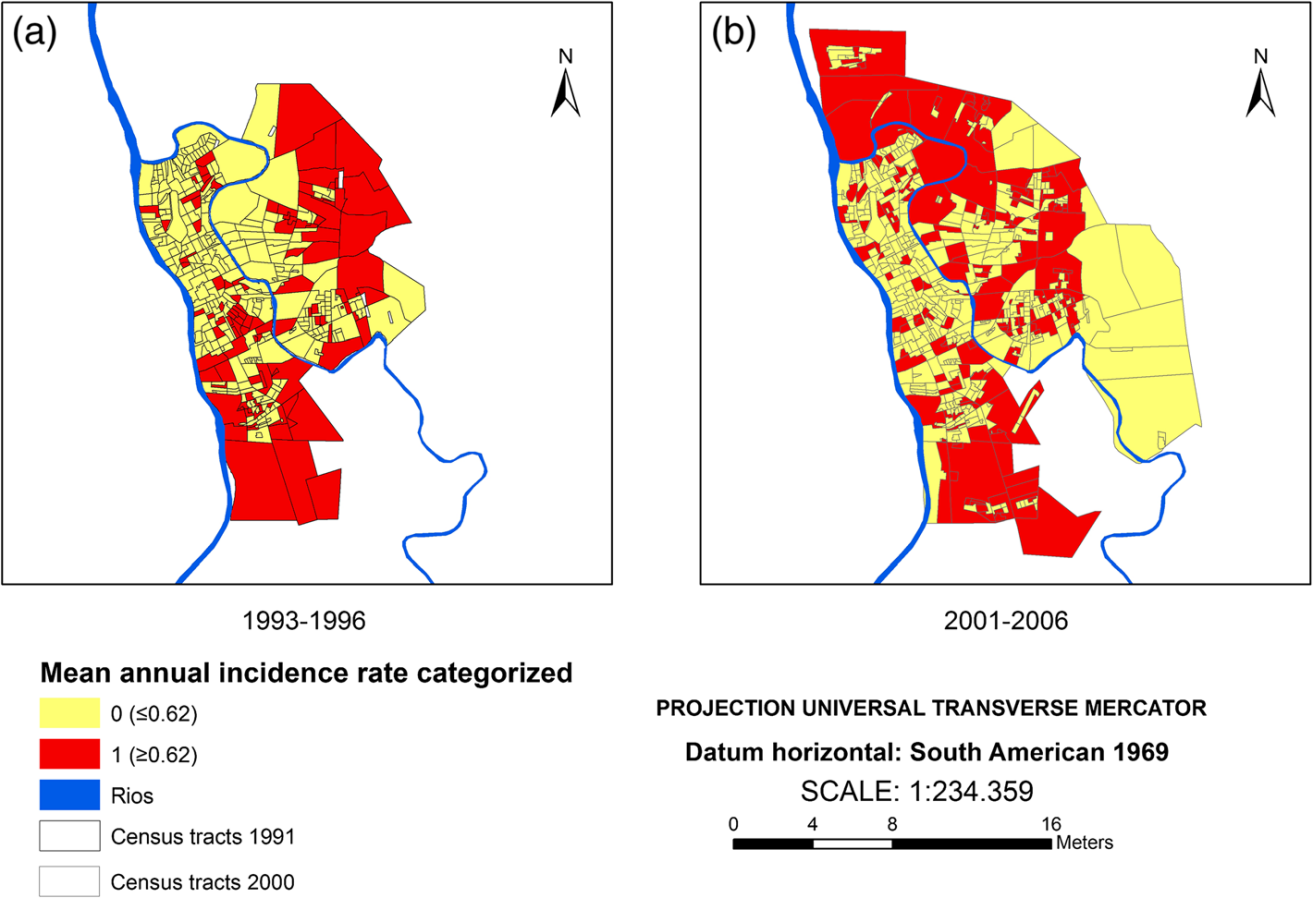
Average annual incidence rate of visceral leishmaniasis in (a) 1993–1996 and in (b) 2001–2006, in the census tracts of Teresina, Piauí, Brazil.

The average annual incidence rates of VL in the CT were calculated for the periods of 1993–1996 and 2000–2006. Census tracts with rates above the 3^rd^ quartile (>0.62 cases per 1000 person-years for the period of 1993–1996 and >0.24 cases per 1000 person-years for the period of 2001–2006) were classified as “high risk” and the ones that did not meet the criteria as “low risk” (Figure 1).

#### Predictor variables

Socioeconomic and demographic variables were obtained from the Demographic Census of 1991 and 2000. The 22 indicators analyzed were categorized according to their quantile distribution, 19 of them were categorized by quartiles, two by the median and one by tertiles (Table 1).

**Table 1:** Socioeconomic indicators selected for analysis

PMENILLITE^1^	Percentage of illiterate men
PPOPILLITE^1^	Percentage of illiterate population
AVERAINCOME^1^	Average nominal income of head of the household
RINCOME^1^	Income ratio - Ratio between total income of upper decile/total income of the poorest 40%
RSEX^1^	Ratio between men population/women population * 100
RDEPEND^1^	Ratio between persons from 0 to 14 and 60 or more years old /15 to 59 years old * 100
PYOUNG5YEARS^1^	Percentage of the population that is younger than 5 years old
PPOORHEAD^1^	Percentage of heads of households with income up to 1/2 Brazilian minimum wage (MW)
PINCOMHEAD3MW^1^	Percentage of heads of households with income up to 3 Brazilian minimum wage (MW)
PILLITEHEAD^1^	Percentage of heads of households that are literate
PILLITEMENHEAD^1^	Percentage of heads of households that are men
PHEADWLESS3^1^	Percentage of heads of households with less than 3 years of schooling
P HEADWLESS7^1^	Percentage of heads of households with less than 7 years of schooling
P3RESHOUSE^1^	Percentage of households with up to 3 residents
P4 RESHOUSE^1^	Percentage of households with up to 4 residents
P5 RESHOUSE^1^	Percentage of households with up to 5 residents
PHOUSEWSAN^1^	Percentage of households without sewage system
PHOUSEWITHSAN^2^	Percentage of households with sewage system connected to the public network
PHOUSEWATER^3^	Percentage of households with water supply connected to the public network
PHOUSEGARBA^2^	Percentage of households with garbage collection
MEANPEOPLE^1^	Mean number of persons per household
R^1^	Rate of population growth

^1^Categorized according to the quartile of the distribution.

^2^Categorized by the median.

^3^Categorized according to the tercile of the distribution.

The environmental classification was obtained through the extraction of land coverage characteristics of Teresina using a Landsat 5 Thematic Mapper (TM) scene of August 1990 and a Landsat 5 Thematic Mapper (TM) scene of June 2003.

### Satellite image classification

Bands 3 (red) and 4 (near-infrared) were used for the image classification, due to larger spectral differences [5].

The image classification, performed with the software *Definiens Developer* 7.0, encompassed two basic steps: multiresolution segmentation and algorithm classification using fuzzy and Boolean logics. This approach uses a combination of spectral, textural, and contextual/topologic information [6].

The rules used to segment and classify the image from 1990 were applied to the image from 2003.

The environmental indicators for both analyzed periods were built by means of calculating the proportion of each thematic class in each census tract (Table 2). This calculation was performed using the program LEGAL in the software SPRING (National Institute of Spatial Research - INPE).

**Table 2:** Environmental indicators selected for analysis

WATER	Proportion of the census tract area covered by water collections (WATER CAT: ≥ 0–10; 10–100)
DENSEVEG	Proportion of the census tract area covered by dense vegetation (DENSEVEG CAT: ≥ 0–1; 1–10; 10–100)
UNDERGROWTH	Proportion of the census tract area covered by pasture and shrubs (UNDERGROWTH CAT: ≥ 0–10; 10–20; 20–100)
DENSEURB	Proportion of the census tract area characterized as residential with little vegetation (DENSEURB CAT: ≥ 0–10; 10–40; 40–80; 80–100)
GREENURB	Proportion of the census tract area characterized as sparse residential with much vegetation (GREENURB CAT: ≥ 0–10; 10–40; 40–90; 90–100)
EXPOSOIL	Proportion of the census tract area covered by bare soil – dirt, mud, sand (EXPOSOIL CAT: ≥ 0–1; 1–2; 2–4; 4–6; 6–10; 10–100)

### Data analysis

The data from the first period (1993–1996) was used as a learning or sample for the development of the predictive model and the second period (2001–2006) as a validation sample.

The CART algorithm (Classification and Regression Trees) was used to attain the predictive model for CTs at high risk for VL occurrence [7]. Generally CART generates a very large tree which shows a minimum number of classification errors, but it is a model excessively adjusted to the data with a limited capacity of generalization. Therefore, the tree has to be reduced (“trimmed”). To get a smaller tree (smaller number of terminal nodes, or “leaves”) it was initially established a restriction of a minimum of 20 observations to be included before a split was attempted and a minimum of 10 observations in a terminal node. Subsequently, by means of the graphic inspection of the relation between the number of terminal nodes of the tree and gains on the classification homogeneity, a tree with 15 nodes was concluded to be most adequate.

To calculate accuracy measures, the cutoff point of 25% to the probability of finding high risk census tracts on the terminal nodes of the tree was used, which simultaneously maximized the sensitivity and specificity, with no important decrease of the global accuracy.

CART models were implemented on the software Splus 4.5. The evaluatiton of the performance of the models was done based on the calculations of the sensitivity, specificity, accuracy and area under ROC curve indicators; estimated in the software Stata 11.0.

## Results

**Figure 2:**
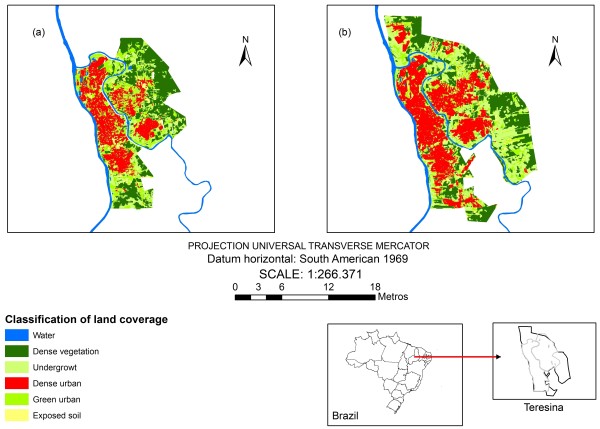
**Classification of land coverage results in Teresina, derived from the image processing of the satellite Landsat 5 TM of 1990 (a) and 2003 (b).** Piauí, Brazil

**Figure 3:**
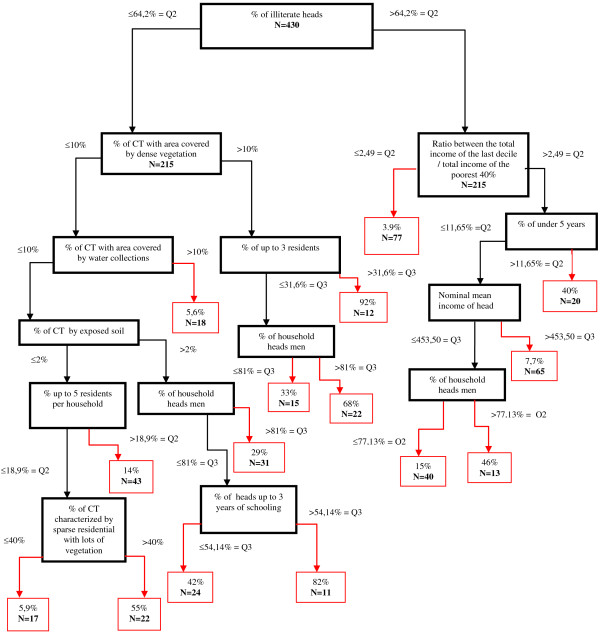
**Diagram of the best predictive model obtained from the learning sample (1993–1996).** The frames in red correspond to terminal nodes with probability (P) and the number of CT (N). Teresina, Piauí, Brazil.

For the period of 1993–1996, more than 70% of the CT reported VL cases, having maximum and median incidence of 4.54 and 0.29 cases per 1000 inhabitants respectively. On the second period analyzed, around 50% of the CT reported VL cases, with maximum and median incidence of 5.91 and zero cases per 1000 inhabitants respectively. The satellite image classification results indicate the expansion of the urban area towards the outskirts of the city, where there was more vegetation coverage (Figure 2).The model developed was capable of discriminating 15 CT sets (Figure 3), with different probabilities of containing CTs at high risk for VL occurrence. This figure shows the 15 sets of CT corresponding to the terminal nodes (in red) and inside each of them the probabilities (P) of the existence of high risk CT and the number of CT (N).

The subset with the lower probability of containing CTs at high risk for VL occurrence (3.9%) included those CTs with the percentage of literate heads of the household higher than the median (>64.2%) and with the income ratio smaller than the median (≤2.49%). The subset with the highest probability of containing CTs at high risk for VL occurrence (92%) encompassed CTs with percentage of literate heads of the household below than the median (≤64.2%), with a larger area covered with dense vegetation, with a percentage of household with up to 3 residents above the third quartile (>31.6%). The other 13 subsets presented the probability of containing CTs at high risk for VL occurrence varying from 5.6% to 82%.

Table 3 shows the model’s performance on the learning (1993–1996) and validation (2001–2006) samples in terms of area under the ROC curve, sensitivity, specificity, accuracy, positive and negative predictive value. The model with 15 terminal nodes (Figure 3) had 79% sensitivity, 74% specificity, 75% global accuracy and 83% area under ROC curve. When applied on the validation sample with the same prediction cutoff (25%), it presented a 52% sensitivity, 66% specificity, 62% global accuracy, and 66% area under the ROC curve (Table 3). ROC curves figures for the validation and learning samples are provided in Additional file 1.

**Table 3:** Predictive performance of the Classification and Regression Tree (CART) model on the learning sample (1993–1996) and validation sample (2001–2006) and their confidence intervals

**Analysis**	**ROC curve area**	**Sensibility**	**Specificity**	**Global accuracy**	**Positive predictive value**	**Negative predictive value**
	**% (95% CI)**	**% (95% CI)**	**% (95% CI)**	**% (95% CI)**	**% (95% CI)**	**% (95% CI)**
**CART (learning sample)**	83	79	74	75	50	92
	(79–88)	(71–87)	(69–78)	(71–79)	(42–58)	(87–95)
**CART (validation)**	66	52	66	62	36	79
	(61–70)	(45–60)	(61–70)	(58–66)	(30–42)	(75–83)

Teresina, Piauí, Brazil.

## Discussion

The results of this study reinforce the notion that the spatial heterogeneity found in the occurrence of the disease is directly related to the living conditions of the population and environmental characteristics of household neighborhood [3]. However, though these relations were possible to be characterized in this study, the validation sample results were not fully satisfactory, indicating that the prediction of areas at high risk for VL incidence is a more complex challenge than the simple identification of associations between environmental and socioeconomic factors and disease incidence, which has been already shown in Brazil and in the world [8-14].

A series of potential explanations for the deficient predictive ability of the model developed could be identified. First, the time interval between both periods analyzed implied in substantial modifications on the dimension and structure of the geographical area under study, including the incorporation on the validation sample of areas that were considered rural in the period of 1993–1996. Second, the data used to derive the predictive model refers to an epidemic period, while the validation sample entails endemic years [12,15]. It is reasonable to suppose that in epidemic situations, due to the typical large magnitude of transmission during these periods, the disease spreads more largely in the geographic space, affecting population subsets that could have eventually been spared in endemic periods. However, having in mind that the VL was only introduced in Teresina in the beginning of the 1980s triggering two epidemics (1981–1985 and 1993–1996), there is still no set of complete data for a typical endemic period that not of 2001–2006. An alternative would be to restrict the analysis strictly to the years between 2001 and 2006, but that would bring difficulties related to the small number of annual cases observed in this period.

Third, the unsatisfactory performance of the model could be due to positive quantitative variations of the socioeconomic indicators, even if the distribution quartiles were used as cutoff points. Since the 1980s in Brazil there has been an improvement on the social and economic indicators. For example, a historical series analysis since the mid-1970s shows a substantial and unequivocal fall of inequality from 2001 to 2004, this last year having the smallest income inequality of the period analyzed [16]. In Piauí the situation is not different, having occurred an increase of 12% of the average income (1985–2006), contributing to the increase in the human development index from 0.57 to 0.70 (1991–2005) and to the reduction of the illiteracy rate from 41.7% to 23.3% (1991–2007) [17,18]. A possible alternative to minimize this problem would be employing a categorization of the indicators by strata of homogeneous areas according to living conditions [19,20].

The VL transmission niches in urban environments not only present a heterogeneous distribution, but also constitute areas with varied landscape and epidemiologic characteristics, where distinct forms of occupation and soil coverage implicate in ecological and social processes which result in huge differences of magnitude on the incidence of the disease.

Therefore, it is possible to infer that the process of establishing and dissemination of VL in the urban environment of Teresina, having a markedly heterogeneous spatial distribution, results from the socio-territorial organization. Nevertheless, we believe that part of the predictive deficiencies of the model is owing to the lack of a better definition of the territory as a spatial-social-environmental unit favorable to VL occurrence.

The improvement on the predictive performance of area at high risk for VL could be attained with the use of more environmental indicators extracted from the satellite images of medium resolution [21,22] or with the use of remote sensing images with high spatial resolution [23]. The satellite images used in this study come from the Landsat Thematic Mapper 5 sensors, which have spatial resolution of 30 meters, in other words, it does not allow the discrimination of elements on the earth’s surface for areas smaller than 900 m^2^. However, VL studies that used medium spatial resolution were able to identify some elements related to vegetation coverage, soil use and patterns of urban occupation associated to the risk of the disease [12,22,24,25], but the difficulties and limitations of these images to properly characterize the local features that determine the pattern of transmission of VL on the urban context are evident [22]. In future studies, high spatial resolution images should be used to better define the local environmental features related to VL occurrence [26].

At last, considering the complexity of the factors involved in the VL dissemination in the urban environment, the results of this study demonstrate that the occurrence of VL on the outskirts of Teresina is intensely related to socioeconomic and environmental problems, arising from the urban expansion process and from the changes in the vector habitat, due to the environmental imbalance caused by deforestation and land occupation with lack of adequate urban infrastructure [25]. In this perspective, focusing interventions in these considered high risk areas could be a useful strategy to improve the effectiveness of control measures while decreasing operational costs.

## Competing interests

The authors declare that they have no competing interests.

## Authors’ contributions

ASA participated in the article’s conceptualization and conducted the literature review, structured the database, analyzed and interpreted the compiled data, and wrote the article. GLW participated in the article’s conceptualization and contributed to the analysis and interpretation of the results and helped write the article. Both authors read and approved the final manuscript.

## Supplementary Material

Additional file 1 ROC curves figures for the validation and learning samples. Click here for file
