# Supplementary material for: Prediction of high-risk areas for visceral leishmaniasis using socioeconomic indicators and remote sensing data
Source: Int J Health Geogr. 2014 May 20;13:13. doi: 10.1186/1476-072X-13-13 (PMC4046095; doi:10.1186/1476-072X-13-13)

ROC curves figures for the validation samples


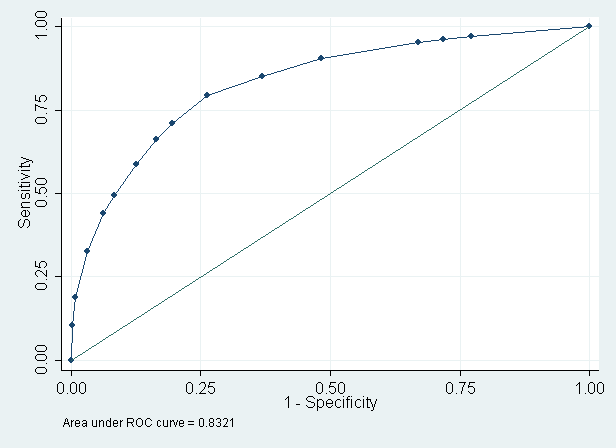


ROC curves figures for the validation samples learning


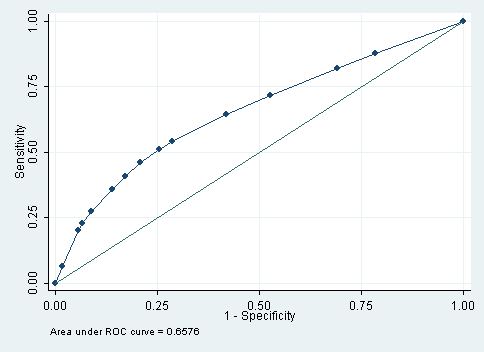

Supplement: Additional file 1 — ROC curves figures for the validation and learning samples. [file 1476-072X-13-13-S1.docx]
